# Small molecules to regulate the GH/IGF1 axis by inhibiting the growth hormone receptor synthesis

**DOI:** 10.3389/fendo.2022.926210

**Published:** 2022-07-28

**Authors:** Lieke M. van der Velden, Peter Maas, Miranda van Amersfoort, Elpetra P M. Timmermans-Sprang, Anneloes Mensinga, Elisabeth van der Vaart, Fabrice Malergue, Henk Viëtor, Patrick W B. Derksen, Judith Klumperman, Andreas van Agthoven, David A. Egan, Jan A. Mol, Ger J. Strous

**Affiliations:** ^1^ Department of Cell Biology, Centre for Molecular Medicine, University Medical Center (UMC) Utrecht, Utrecht, Netherlands; ^2^ Specs Compound Handling, Zoetermeer, Netherlands; ^3^ Department of Pathology, University Medical Center (UMC) Utrecht, Utrecht, Netherlands; ^4^ Department of Clinical Sciences, Faculty of Veterinary Medicine, Utrecht University, Utrecht, Netherlands; ^5^ Department of Research and Development, Beckman Coulter Life Science, Immunotech Marseille, Marseille, France; ^6^ Drug Discovery Factory (DDF) Ventures, Breukelen, Netherlands; ^7^ Cell Screening Core, Department of Cell Biology, Center for Molecular Medicine, University Medical Center, Utrecht, Netherlands

**Keywords:** GH inhibitor, IGF1 inhibitor, cancer, autocrine, ribosomal synthesis, BM001

## Abstract

Growth hormone (GH) and insulin‐like growth factor‐1 (IGF1) play an important role in mammalian development, cell proliferation and lifespan. Especially in cases of tumor growth there is an urgent need to control the GH/IGF1 axis. In this study we screened a 38,480-compound library, and in two consecutive rounds of analogues selection, we identified active lead compounds based on the following criteria: inhibition the GH receptor (GHR) activity and its downstream effectors Jak2 and STAT5, and inhibition of growth of breast and colon cancer cells. The most active small molecule (BM001) inhibited both the GH/IGF1 axis and cell proliferation with an IC50 of 10‐30 nM of human cancer cells. BM001 depleted GHR in human lymphoblasts. In preclinical xenografted experiments, BM001 showed a strong decrease in tumor volume in mice transplanted with MDA‐MB‐231 breast cancer cells. Mechanistically, the drug acts on the synthesis of the GHR. Our findings open the possibility to inhibit the GH/IGF1 axis with a small molecule.

## Introduction

Throughout history, since the biblical story of David and Goliath, gigantic people have drawn interest. In 1886 Pierre Marie named the condition of non-congenital singular hypertrophy of the upper, lower and cephalic extremities “acromégalie”. In 1912 Harvey Cushing associated both short stature and increased growth with structural changes in the pituitary gland and postulated that there was a particular “hormone of growth”. The history of growth hormone (GH) has been elegantly described by Buchman, Bell and Kopchick (1). The state-of-the-art of the GH field has been reviewed in (2–5). GH receptor (GHR) acts as a modulator of cellular metabolism, whose loss is not lethal, but results in sub-optimal health with short stature, decreased bone mineral density, decreased muscle strength, thin skin and hair, increased adiposity, and hepatic steatosis. Interestingly, short people without GH signaling live normal lives, but are highly resistant to cancer and diabetes type 2. In a well-studied Ecuadorian cohort of 100 persons, no cancer deaths were observed (6). They also performed significantly better in memory tasks and had lower cognitive impairment compared to their unaffected relatives (7).

GH exerts its functions by binding to the GHR on target cells, which activates the Jak/STAT/Src pathways and stimulates the production and secretion of IGF1 in many tissues, mainly the liver. In the brain, where it plays an important role in development and function, IGF1 is GH-independent (3, 8–10). Since IGF1 was identified in 1957, the interest increased, especially when IGF1 was found to mediate the anabolic and mitogenic activities of GH (11). IGF1 is a 70 amino acid peptide with a molecular weight of 7,649 Da. In addition to its cognate receptor, the type I IGF1 receptor, it has a low affinity to the insulin receptor. While GH is short-lived, IGF1 has a long half-life in the blood of 16-24 h. GH and IGF1 both stimulate linear growth but GH is more efficient. IGF1 has a GH-independent growth stimulating effects that in some cases acts synergistically with GH (12), while GHR signaling has growth promoting effects, independent of IGF1.

Given the ability of GH and IGF1 to promote cell proliferation, cell movement and angiogenesis, and to suppress apoptosis, it is not surprising that dysregulation of the GH–IGF1 axis promotes neoplasia in human (13, 14). The evidence points to a role of GH/IGF1 in stem cell niches, where hormones from surrounding stroma, among others GH/IGF1, address stem cells. In such an environment increased concentrations of GH/IGF1 might stimulate early stages of cancer cell growth (15, 16). A prevalent hypothesis is that GH stimulates cancer stem cell growth, while GH/IGF1 drives epithelial-to-mesenchymal transition and metastasis (17–21). In addition to the pituitary, GH is expressed in most cancer cells, most notably in colon, prostate, breast, lung, osteosarcoma, and melanoma cells (22–26).

A growing number of studies indicate the involvement of *autocrine* GH and IGF1 in tumor growth promotion, and demonstrate that effective therapeutic options for cancer treatment need to drastically lower serum IGF1, reviewed in (13):. Attempts to successfully develop effective IGF1 inhibitors have failed, as IGF1 acts in an autoinhibitory loop with GH in the pituitary gland: interrupted IGF1 signaling causes upregulation of the Jak/STAT pathway *via* high GH concentrations in the blood. Early attempts to inhibit GH secretion to treat breast cancer include hypophysectomy (27) and application of somatostatin receptor ligands, such as octreotide, lanreotide, pasireotide, somatoprim (28). Treatment with the GH antagonist, Pegvisomant, is currently the only way to reduce GH activity (2, 29), but this will only inhibit cell membrane presented GHR but not autocrine intracellular signaling in cells co-expressing GH and GHR.

Herein we describe the identification of the first small molecular inhibitors of the integral GH/IGF1 axis. The molecules act on the synthesis of the GHR and deplete cells from both endocrine and autocrine GH signaling activity. Consequently, mice treated with the molecules have low IGF1 serum levels. In addition, the molecules inhibit the growth of triple-negative breast cancer cells both in tissue culture and in a xenografted mouse model of breast cancer.

## Materials and methods

### Cells, plasmids transfections, antibodies, reagents microscopy

For the fluorometer assay hGH and hGM-CSF were purchased from R&D system. Anti-Phospho STAT5-PE conjugate (Tyr694, clone C71E5) is from CST. Inhibitory controls, BMS-911543 and INCB018424 (Ruxolitinib), were purchased from Chemietek, Indianapolis. Human embryonic kidney 293 (Hek293) cells, were used as described previously (30), and were maintained in DMEM high glucose (4.5 g/l), supplemented with 10% FCS, 100 U/ml penicillin, 100 mg/ml streptomycin and 600 mg/ml G418 as described previously (31). IM9 cells were maintained in RPMI 1640, 10% FCS, penicillin, streptomycin, containing 4.5 g/l glucose and 1 mM sodium pyruvate. γ2A cells were maintained in DMEM low glucose (1.0 g/l), supplemented with 10% FCS, 100 U/ml penicillin, 100 mg/ml streptomycin. Stable γ2A cell lines with Jak2 in pDONR201 were maintained in medium, supplemented with puromycin (1 mg/ml). The expression of Jak2 was induced by addition of 1 mg/ml doxycycline (Clontech) 24 h before the experiment. MM231 and Colo-205 cells were grown in DMEM containing 10% FBS. Human GH was added at a concentration of 180 ng/ml. DNA transfections were performed using FuGene 6 (Roche, Applied Sciences) according to the manufacturer’s instructions. N-FLAG-tagged wild-type mouse JAK2 constructs were a generous gift of Prof. Carter-Su (University of Michigan, Ann Arbor, MI). Jak2 and its binding Y119E-mutant were cloned into pDONR201 (Life Technologies) for stable expression in γ2A cells. pDONR201 was a kind gift of Dr. Puck Knipscheer, Hubrecht Institute, Utrecht. The fos-GHRct DNA construct in pcDNA3 was described previously (32). Antiserum against human GH was raised in rabbits. Mouse anti-phosphotyrosine antibody was from Millipore (clone 4G10), anti-HA tag antibody (clone 12CA5) was from BAbCO (Richmond, CA), and anti-FLAG tag M2 antibody was from Sigma. Rabbit anti-GHR B antibody was described previously (33, 34). Monoclonal antibody against phosphorylated Tyr-1007 and Tyr-1008 of JAK2 was from Abcam (ab32101). Alexa Fluor 680- and IRDye 800-conjugated goat anti-mouse and anti-rabbit IgGs were obtained from Molecular Probes. GHR-expressing HEK293 cells grown as described (30) were transfected with FuGENE 6 (Roche Applied Science) according to standard conditions or with the calcium phosphate method. Microscopy studies were performed as previously described (31).

### Assays

Drug handling and cell-based assays are detailed in **Supplementary Data 1**, automation. High-Content StratoMineR (35). After incubation with the compounds, the cells were fixed and the fluorescence (anti-GHR, anti-pY, anti-pJak2, anti-pSTAT5) was quantitated.

### Flow cytometric test

First round: The PerFix EXPOSE (Beckman Coulter), was used to fix and permeabilize cells according to the optimized procedure described in Malergue et al., 2015 (36). Samples were analyzed in FC500 MPL. Data were analyzed with Kaluza software (Beckman Coulter). Second round: the PerFix EXPOSE was used according to the IFU and samples were analyzed in a Navios Cytometer. The first screening used pure IM-9 cells, whereas in the second round they were spiked in 100 µL whole blood (EDTA tubes). 15 min after the inhibitory compounds, the activators were added for 20-30 min.

### Lysis and immunoprecipitations

For GHR-Jak2 co-immunoprecipitations, the cells were lysed in 20 mM Tris pH 8.0, 150 mM NaCl, 0.5% NP40, 1 mM PMSF, 10 mg/ml aprotinin, 10 mg/ml leupeptin. Cell lysates were centrifuged to pellet the nuclei and the supernatants were used for GHR isolation in 1% Triton X-100, 0.5% SDS,0.25% sodium deoxycholate, 0.5% BSA, and inhibitors *via* immune precipitation with anti-GHR and protein A beads. Immunoprecipitates were subjected to reducing SDS-PAGE and transferred to Immobilon-FL polyvinylidenedifluoride membrane (Millipore). Blots were immunostained with the indicated primary antibodies followed by Alexa Fluor 680, Alexa-800 IRDye conjugated anti-mouse or anti-rabbit antibodies. Detection was performed with an Odyssey system (LI-COR Biosciences).

### *In vitro* translation

pcDNA3 vector DNA expressing Fos-zippered GHR cytosolic tails as described in (32) was used in a cell-free translation system, based on HeLa cell extract (Promega). Empty vector (pcDNA) was used as control. The blots were detected with an anti-GHR antibody raised in rabbits against GHR cytosolic tails.

### Animal experiments

Female RAG2^-/-^; IL-2Rgc^-/-^ immune deficient mice (37) were purchased from Envigo (Horst, The Netherlands). Orthotopic transplantations, longitudinal tumor measurements and bioluminescence imaging were performed as described (38). Mice were treated when tumors reached a volume of >50 mm^3^, three times weekly for a period of three weeks with the indicated drug concentrations. Animals were euthanized when tumor volumes exceeded 200 mm^3^ or when bioluminescence imaging revealed metastases.

### Chemistry, drug characterization

The compound library was provided by Specs (Zoetermeer, The Netherlands). The library was composed of 38,480 small druglike molecular compounds and was stored in dimethyl sulfoxide (DMSO) at 10 mM per compound in 96-well plates at −20°C. After visually inspections and Quality control by LC/MS on a sample of the plates, they were reformatted in 386 plates. Analogues of the hits were selected by substructure and similarity searching using ISIS/base software (Accelrys, San Diego, CA, USA) in two consecutive rounds. Analogues were also provided in DMSO at 10 mM per compound in 96-well plates at −20°C. Resupplies for further testing were provided as dry powder. More details are in **Supplementary Data 1**, Chapter 1.

## Results

### The drug discovery protocol

**Figure 1:**
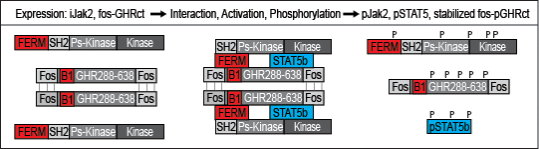
*Diagram of the primary screen*. Depicted are fos-GHRct (amino acid 288-638 with fos zippers (fos), box-1 sequence (B1)), and Jak2 (FERM, SH2, pseudokinase and kinase domains). If fos-GHRct and Jak2 bind *via* FERM and box-1, Jak2 gets activated and phosphorylates itself, the dimerized fos-GHRct and STAT5b. After phosphorylation of fos-GHRct, Jak2 and STAT5, the complex dissociates. As fos-pGHRct is protease-resistant, it accumulates in the cells. Unphosphorylated fos-GHRct is rapidly degraded. Steady state levels of fos-pGHRct are lower if one of the 6 scenarios is valid. The cell-based assay was performed in γ2A cells carrying inducible Jak2 (iJak2) or binding-deficient iJak2 Y119E (as control) (**Figure 2**).

**Figure 2:**
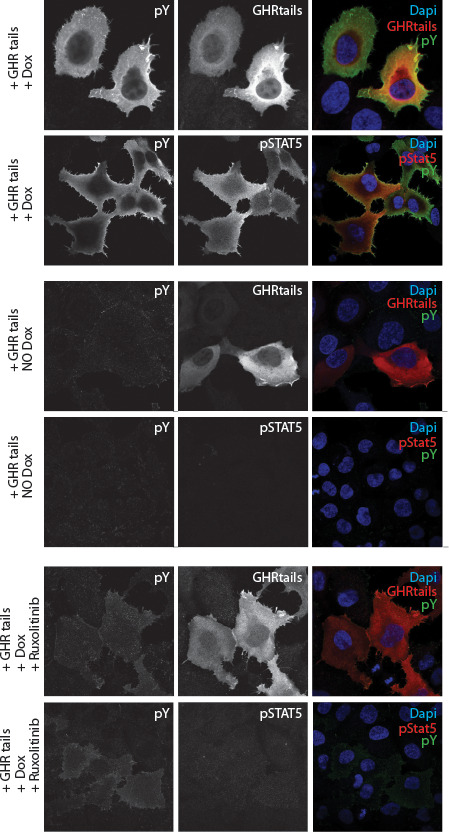
*Immune fluorescence staining of human y2a cell line stably transfected with an inducible Jak2 expression construct.* Transient transfected zippered fos-GHRct accumulate only in the presence of Jak2 and induce phosphorylation of the tails, of Jak2, and STAT5 upon doxycycline-induction of Jak2 synthesis.

As it was unknown whether the GH/IGF1 axis is druggable, we designed a broad strategy based on our previous studies in which soluble fos-zipped GHR cytosolic tails (fos-GHRct) can serve as signaling complexes if co-expressed with Jak (32, 39). Fos-GHRct expressed without Jak2 is immediately degraded by the ubiquitin pathway. Phosphorylated fos-GHRct (fos-pGHRct) detaches from Jak2 and unzips. Only unzipped fos-pGHRct are protected from proteasomal degradation and accumulate in the cell. This system offers a sensitive assay with functional parameters: anti-GHR (a) anti–pY (b), anti-pY Jak2 (c) and anti-pY STAT5b (d) (**Figure 1**). For 6 scenarios the signal will be decreased: (1) inhibition of Jak2 kinase activity, (2) inhibition of fos-GHRct expression, (3) inhibition of the fos-GHRct : Jak2 interaction, (4) increased degradation of fos-pGHRct, (5) increased degradation of Jak2, and (6) dephosphorylation of fos-pGHRct (32, 39). As detailed in **Supplementary Data 1**, chapter 1, **Figure 2** shows the specificity of the immune fluorescence. As control we used 10 µM Ruxolitinib to inhibit Jak activity, and iJak2-119E cells that express binding-deficient Jak2 (not shown).

A 38,480-small molecule library was supplied by Specs Compound Handling BV, Zoetermeer, nearly identical to libraries I and II combined as used in (40). Purity of the compounds was verified (>90%), and the library was formatted to 384-well plates. The details of the screening results are in the supplementary data (**Supplementary Data 1**, Chapter 1).

**Figure 3:**
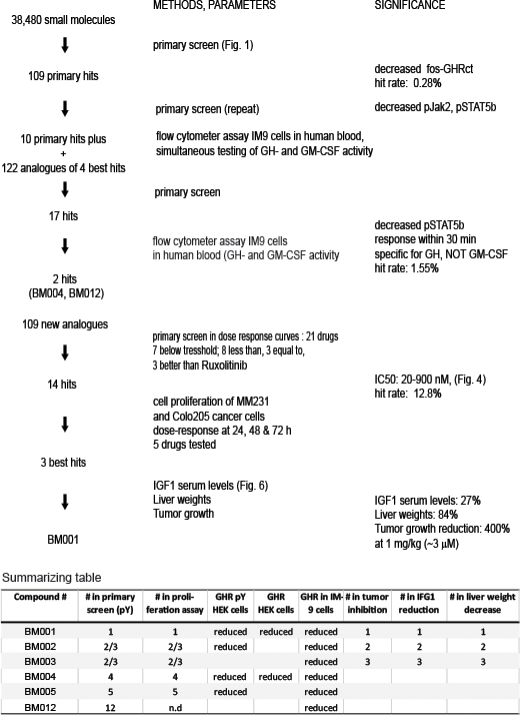
*Drug discovery scheme of GH/IGF1 axis inhibitors with the key steps*. The last three columns of the summarizing table are explained in **Figure 4**.

The drug discovery scheme is delineated in **Figure 3**. Based on the normalized data of the pY and GHR signals we selected 109 compounds that decreased the presence of fos-pGHRct (hit rate 0.28%, thresholds defined in **Supplementary Data 1**). The best 10 were supplemented with 122 analogous compounds from Specs (**Supplementary Data 2**), and tested in the primary screen. The best 17 compounds, based on activity and chemical representation of the different hit compounds, were tested in a flow cytometer-based assay of whole human blood, supplemented with human IM-9 lymphoblasts (36). This allowed simultaneous testing of GH- and GM-CSF activity (described in **Supplementary Data 1**, Chapter 4). The GH/GM-CSF cytometric test added two important criteria to GH signaling: Compounds that exert a rapid effect on the GH/IGF-axis are prioritized as the response time for STAT5 phosphorylation was limited to 20-30 min. Also, it selected compounds that target the GH-driven signaling at the expense of another Jak2-dependent cytokine signaling receptor (GM-CSF). Given the short resident time of the GHR both in the synthesis (ER) and the signaling compartment (PM) of less than 30 min, candidate hits must have an immediate and strong effect on the signaling capacity *via* the plasma membrane. GH-induced GH-GHR-Jak2 signaling was probed by GHR and pY staining in concentration series (0-20 µM, 20 h drug-treated, 10 min GH) (**Figure S11**). Based on all data combined with the GH/GM-CSF cytometric (FACS) test, we selected 2 hits, BM004, BM012 (hit rate 1.55%, **Figure S8**, **Table S1**). Both compounds lowered the phosphorylation of fos-pGHRct, of the active site of Jak2 (p1007/1008) and of STAT5 (pSTAT5) (**Figure S10**) and were inactive towards GM-CSF signaling (**Figure S8B**). One compound (BM004) showed the same potential (IC50 ~ 0.2 µM) as Ruxolitinib (IC50, 0.1-0.5 μM) (41). Both lead compounds reduced the levels of tyrosine phosphorylated full length GHR, stimulated with GH, concentration-dependent in GHR-transfected Hek293 cells (**Figure S11**).

**Figure 4:**
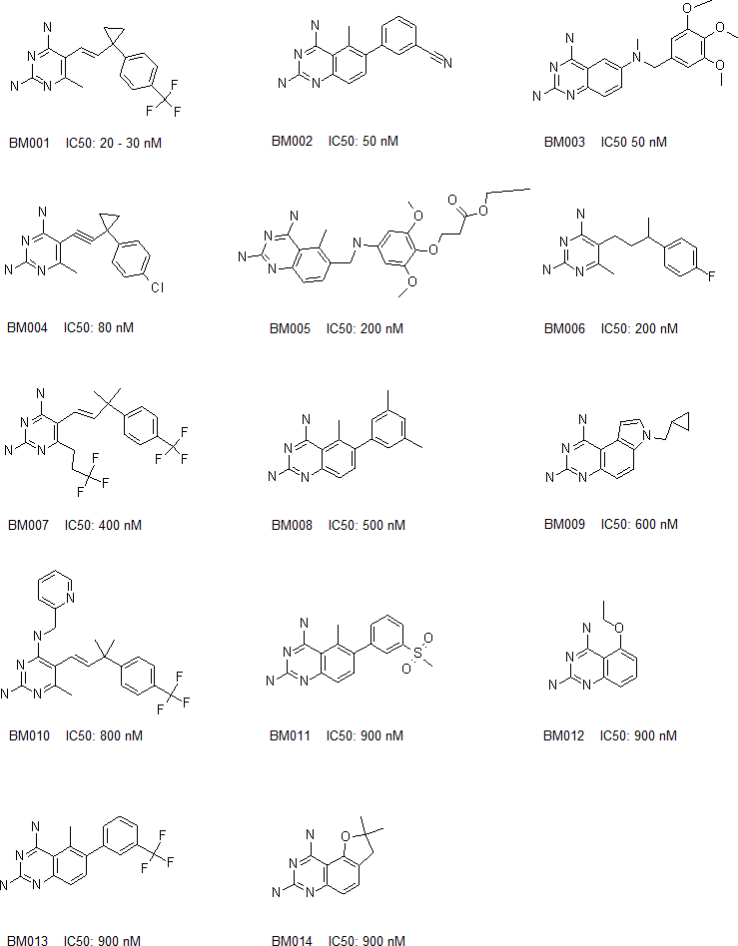
Chemical structures, ranked according their inhibitory effects on GHR signaling and/or cell proliferation.

**Figure 5:**
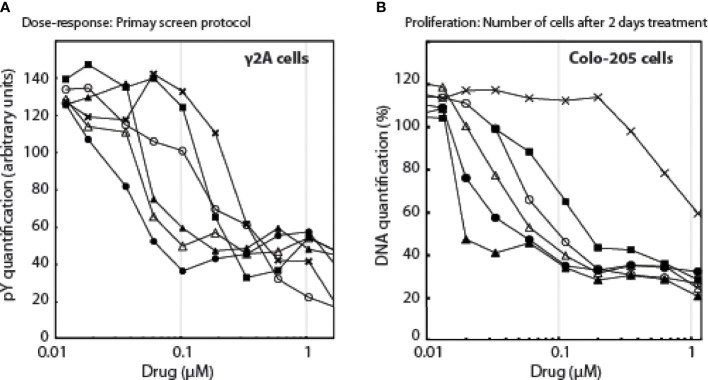
The effect of the drugs as a function of drug concentrations, **(A)**: on pY signals, **(B)**: on the number of cells.Λ, BM001; π, BM002; Δ, BM003; ν, BM004; X, BM005; ○, Rux.

Based on these 2 compounds, Specs selected a new set of 109 structural analogues (**Supplementary Data 3**). Twenty-one compounds tested positive in the primary screen protocol. In concentration curves 7 compounds were below threshold, 8 were less, 3 similar and 3 were better than Ruxolitinib. (**Figures S12, S13**). The 14 active drugs are depicted in **Figure 4**. In the final test, the 5 best compounds (BM001-BM005) were screened for their proliferative effect on cancer cells. We used the triple-negative human breast cancer line, MDA-MM-231 (MM231) and the human colon cancer line Colo-205, and quantified DNA in vital cells (**Figure 5B**). Dose-response curves were made after 24, 48 & 72 h. The 5 drugs inhibited both cell lines relatively equal to the dose-response curves monitoring pGHR tails, pJak2, and pSTAT5 (**Figure S13** and **Figure S14**) with IC50 values varying from 30-200 nM (**Figure 5**).

### The drugs in a preclinical *in vivo* model

**Figure 6:**
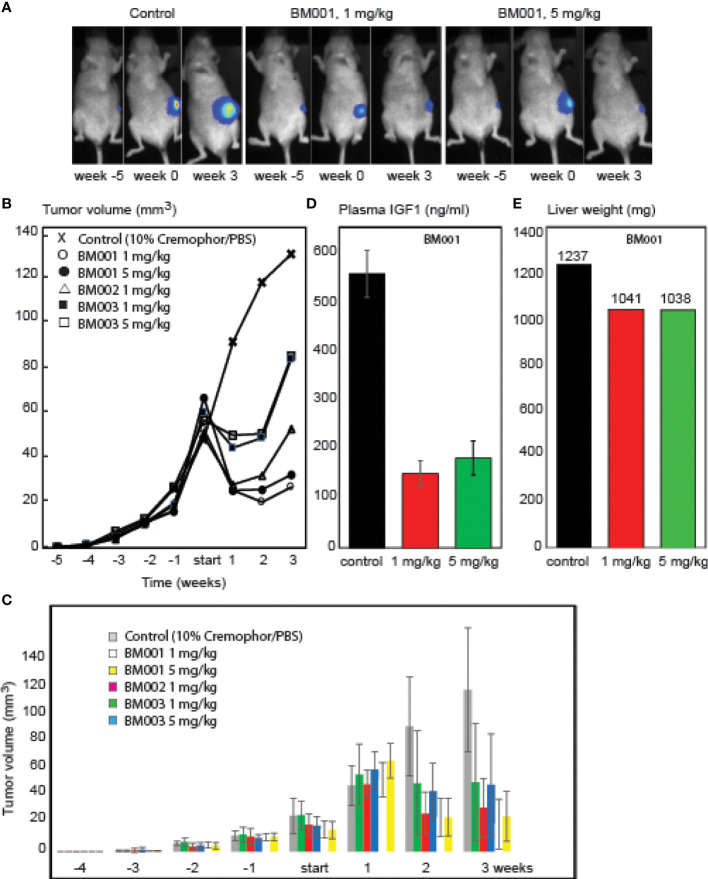
*Effect of the identified GHR drugs on breast cancer growth in mice.* A. Compounds BM001, BM002 and BM003 inhibit tumor growth. Bioluminescence imaging was used to visualize tumor growth over time **(A)** and tumor volumes were longitudinally measured in response to the indicated BM compounds **(B, C)**. BM001 treatment results in a dose-dependent reduction in plasma IGF1 levels **(D)** and liver weight **(E)**. Statistical analysis was performed with GraphPad Prism 8.0 (San Diego, CA, USA). Data is presented as mean SEM.

BM001, BM002, and BM003 were used in mice xenografted with MM231 breast cancer cells that are GH-responsive both for growth and drug resistance (42–45). The cells were orthotopically grafted into the inguinal mammary gland, and drug treatments started when tumors reached a volume of >50 mm^3^. We used a treatment regimen of 3 injections per week (1 and 5 mg/kg) during 3 weeks (9 mice/group) (**Figure 6**). Animals were killed when tumor volume exceeded 150 mm^3^ or mice presented lung metastasis upon bioluminescence imaging measurements. All 3 drugs had an immediate effect on the tumors: growth halted and the tumor sizes were reduced. While tumor volumes in mice treated with the less potent drugs (BM002 and BM003) started to increase again, BM001 treatment continued to suppress tumor growth, even at the lowest concentration (**Figures 6A-C**). Drug withdrawal effects were not studied until now. Recent experiments show that prolonged treatment continues to suppress tumor growth. We observed no adverse side effects neither on appetite and behavior (46). Pathological examination of epithelia did not reveal any damage or abnormalities. We next assessed if the identified drugs indeed target the GH/IGF1 pathway and consequently, if they result in a decrease in liver weight and IGF1 levels, as observed in GHR knockout mice and Laron patients (47). Indeed, we observed that the most effective compound, BM001, reduced serum IGF1 levels (73%) and liver weight (16%), while total body weights remained the same (**Figures 6D, E**). The results of **Figure 6** add another important piece of information: they show that in addition to the human and rabbit GHR system (rabbit-derived cDNA was used for the primary screen, **Figure 1**), BM001 impacts systemically on the GH/IGF1 axis in mice.

### At the molecular level

**Figure 7:**
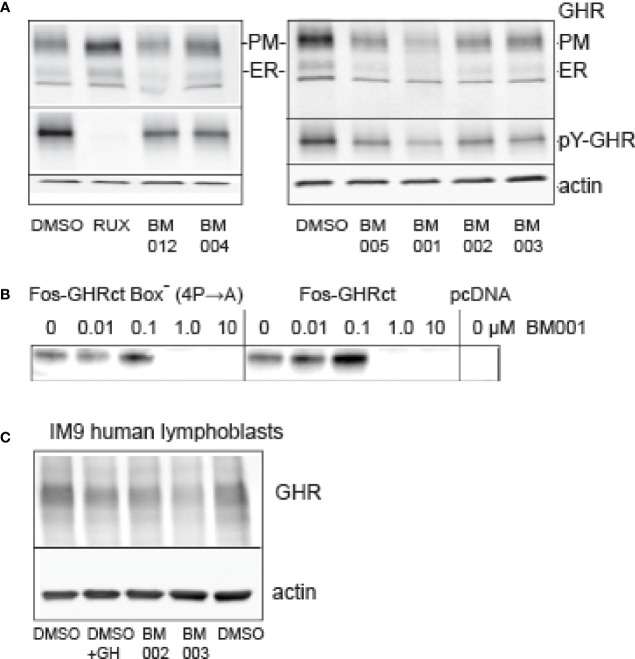
*Effect of drugs on protein synthesis.* **(A)**: Effect of compounds on GHR. Hek293T cells, transfected with GHR, were treated overnight with 10 µM drugs as indicated (RUX, Ruxolitinib). The amounts of GHR both at the plasma membrane (PM) and in the ER (ER) were decreased in the drug-treated cells. BM001 had the strongest effect. The cells were treated for GH for 10 min before lysis to be able to monitor the pY signals, after GHR isolation by immunoprecipitation (middle panels). The effect of the drugs on the pY signals reflected those of the GHR levels (only PM species were visible). In the presence of Ruxolitinib, the protein levels were unchanged, while the pY label (middle panel) was absent. The lower panel (actin) shows that the amounts of cells were identical across the experiment. This experiment represents the results of 6 independent repeats. **(B)**: BM001 concentration effects on the synthesis of fos-GHRct in a HeLa cell cell-free system. Synthesis was inhibited irrespective of the presence of a functional box-1 sequence (IC50 0.1-1.0 µM). In the right panel the 4 proline residues in box-1 were replaced by alanine residues. Empty vector (pcDNA) was used as control. The blots were detected with anti-GHR(B). The *in vitro* translation was performed 5 times that all show a clear inhibitory effect of BM001 on the GHR signal. **(C)**: Effect of selected drugs on GHR levels in human lymphoblasts. IM‐9 human B lymphoblasts express endogenous GHR. Lymphoblasts were incubated with the drugs for 4 hrs or with GH for 1 hr. GHRs were isolated by immunoprecipitation. Drugs BM002 and BM003 reduce the levels of GHR to ~60% in 4 h. This experiment is representative of 6 independent experiments.

In the primary screen we used soluble fos-zipped GHR cytosolic tails that act in signaling complexes if co-expressed with Jak2. Initially, 6 scenarios were anticipated that can lower these signals. In the discovery itinerary we used the flow cytometer assay twice to find drugs that act on the GH-induced Jak2/STAT5 pathway and not on the identical GM/CSF-driven pathway (**Figure 3**, **Figure S8**). As the GM/CSF-induced Jak2/STAT5 pathway was not affected by the 2 hit compounds (BM004 and BM012), that served as prototypes for the final analogue selection, we excluded the possibility that they act on the Jak2 signaling pathway directly (scenario 1). To test scenario 2 (inhibition of fos-GHRct expression), we treated GHR-expressing Hek293T cells with a selection of drugs from the cell proliferation studies and analyzed the GHR species. **Figure 7A** shows that both the immature GHR in the endoplasmic reticulum (ER), and the mature GHR at the plasma membrane (PM), diminished with the drugs. Activation with GH before lysis enabled estimation of the pY signals on GHR; it paralleled the GHR blotting, controlled by Ruxolitinib. The strongest effect was obtained with BM001. The ratio between the amounts of GHR in the ER and at the PM reflects a steady state of synthesis (ER) and transport to the PM followed by endocytosis and degradation in the lysosomes. If the compounds inhibit transport from the ER to the PM, GHR would accumulate as the lower molecular (ER) species; if they inhibit endocytosis and/or degradation, GHR would accumulate as the higher molecular PM species. Because both species diminished, the drugs either inhibit the synthesis of GHR molecules or induce rapid GHR degradation in the ER. To decide between these possibilities, we performed *in vitro* translation experiments. BM001 effectively inhibits the synthesis of fos-GHRct (0.1-1 µM, **Figure 7B**, right panel). To exclude scenario 3 (inhibition of the GHR : Jak2 interaction) we replaced the proline residues in box-1 (the binding site for Jak2) with alanine residues. **Figure 7B**, left panel, shows no change compared to an intact box1, indicating that the GHR : Jak2 interaction is not involved in the inhibitory effect of the compounds. These findings render scenarios 4-6 (increased degradation of fos-pGHRct, increased degradation of Jak2, and dephosphorylation of fos-pGHRct) unlikely. To show that the drugs also work on the levels of endogenous GHR we used IM9 human lymphoblast cells that express easily detectable amounts of GHRs (48).

## Discussion

In this study we describe 14 molecules that inhibit the synthesis of soluble fos-zipped GHR cytosolic tails. The best 5 drugs inhibited breast and colon cancer proliferation with IC50 ranging between 20-200 nM. Detailed selection on cell proliferation and synthesis inhibition narrowed the selection to 3 compounds that were tested on tumor growth inhibition in mice. A convincing feature was that their relative potency of inhibiting tumor growth coincided with their potency to decrease IGF1 levels and liver weight (**Figure 3**, summarizing table, **Figure 6**). This indicates that they act *via* the same mechanism. A second important feature is that these 3 compounds act on cell growth at a favorably low IC50, varying between 10-50 nM.

Inhibiting *the GHR synthesis* is most attractive for designing a drug to control the GH/IGF1 axis. It closely resembles GHR knock out mice, and enables *measured* GHR function disruption, unlike *complete* inhibition in Laron patients (6, 49, 50). A second requirement for a useful drug is that it acts both fast and long-lasting. This is not obvious for the GH/IGF1 axis: unlike most growth factor receptors, the GHR is continuously synthesized and degraded with a half-life of 30-60 min (51–53). Also, GH has a very short resident time in the circulation. Conversely, IGF1, the most important down-stream effector, has a long *in vivo* half-life of 16-24 h. Flow cytometer-based assay with IM9 cells in human blood confirmed the rapidity of action: the drugs decreased the phosphorylation of STAT5b within 30 min. To address the effectivity question, we injected the mice once every 2-3 days, i.e. with 2-3 times longer intervals than the resident time of IGF1 in serum. Low GH signaling as well as low IGF1 levels generate high GH serum levels due to lack of negative feedback inhibition (54). Under these conditions, even low levels of GHRs would be able to reactivate IGF1 gene expression in the liver. Generally, the effect of GH on the increase of plasma IGF1 concentration is rather slow. This is in part also due to a slow increase in IGFBP3. BM001 reduces IGF1 four-fold as measured at the end of the experiment together with a decrease in liver weight. This implies that the drug acts effectively with a long-lasting effect on IGF1 synthesis. Unfortunately, we could not demonstrate increased GH concentrations in the xenografted mice with our GH assay (not shown). GH levels were measured after the mice were sacrificed, 4 h after the last drugs injection. The dynamics of drug serum levels and GH half-life under these conditions need more study to reliably define the effect of the drugs on serum GH. Alternatively, suppression of GHR activity for three weeks might have blunted the GH secretion, resulting in lower liver weights. As the total body weight did not decrease, an effect of IGF1 decrease due to lower food intake seems unlikely. Recently, we checked glucose and insulin levels in a dog experiment: BM001 did neither affect glucose nor insulin (data not shown). At the molecular level, we showed that the drugs rapidly deplete both the endoplasmic reticulum and the plasma membrane from GHR species (**Figure 7A**). Also, the amounts of GHR tails, synthesized on free polyribosomes both in an *in vitro* system (**Figure 7B**) as in the intact cells (**Figure 5A**, and primary screen) were decreased in the presence of the drugs. Most likely, the drugs act at the level of ribosomal synthesis. An alternative explanation, drug-induced degradation, is less likely as it implies proteolytic degradation by two different proteolytic systems of the same substrates: the cytosolic ubiquitin system and ER quality control. In addition, the drugs do not interfere with rapid depletion of the GHR from the plasma membrane (53, 55).

Our finding generates an important tool to answer the question of how the GH/IGF1 axis drives the growth of cancer cells. Tumor tissue from major cancer types as well as cells derived from them express both GH and GHR. Although GHR is expressed in virtually all cells of the body, especially melanoma, breast, lung, ovarian and prostate, cancers are high expressors (56). MM231 cells, used in this study, generate GHR mRNA-dependent resistance to apoptosis induced by chemotherapeutic drugs (44, 45, 57, 58). In cultured cancer cells, there is no direct link between GHR mRNA levels and growth rate. Added to the finding of Barclay et al. that basal GHR expression level is 4.8-fold higher, comparing GHR Box1-/- vs. GHR-/- (59), GHR expression levels are probably not the determining factor behind cancer growth. Slowly but surely, evidence is accumulating that autocrine expression of GH is required for accelerated growth of tumor cancer cells. A key element in this mechanism might be that GH expression generates a constantly activated GHR, starting from the Golgi complex (60).

Downstream in the GH/IGF1 signaling there is an strong analogy between inhibiting the GHR in mice with BM001 (**Figure 6B**) and the inhibitory effect of the small molecule S3I-201 on STAT3 with the same cancer cells (61, 62): Both inhibitors resulted in tumor regression of MM231 xenografted mice. In breast cancer cells STAT3 is constitutively activated (63). In fibroblasts from children with an abnormal GH-dependent STAT3 activation, cell cycle arrest occurred in G0/G1 (64). This suggests that, with no indication of increased apoptosis, the drugs might act *via* STAT3 to induce cell cycle arrest. How the drugs act on cell cycle regulation is of interest for treatment protocols. If the drugs terminate cancer growth by forcing the cancer stem cells into senescence a relative short treatment might be sufficient to cure the cancer as the tumor will shrink due to lack of fresh cells. If the drugs only keep the cancer stem cells in G0/G1, longer periods of treatment are probably needed to prevent recurrence. An elegant study with spontaneous dwarf rats which lack GH and have very low IGF1 levels shows that the growth of N-methyl-N-nitrosourea-induced mammary tumors fully depends on GH injections. Tumor regression occurs upon GH withdrawal. STAT3 phosphorylation is fully in line with this protocol and is not detectable in regressing tumors (65). When after one month, GH injections were resumed, the tumors re-emerged at their original positions, suggesting that these original tumors were not completely extirpated by the lack of GH.

Use of small molecules to specifically inhibit a cytokine receptor is a major challenge: the most obvious specificity is in its interaction with members of the Jak tyrosine kinase family *via* the box-1-FERM interface (66). Although there is clear insight in the functionality of this mechanism (67), the application of small molecules to effectively interfere with this transient type of protein-protein interaction is difficult due to the low affinity (32) and the similarity among the box-1 sequences of the many cytokine receptors. In addition, the molecular machinery involved in the cytokine receptor signal transduction and degradation bears considerable analogy (68, 69). Therefore, efforts to inhibit cytokine action in various diseases have focused on Jak/STAT activity with some success. Jak-STAT dysregulating pharmaceuticals are used to fight autoimmune disorders such as rheumatoid arthritis, ulcerative colitis, Crohn disease, myelofibrosis, polycythemia *vera*, and other myeloproliferative diseases (70, 71). Currently, the only method to specifically regulate GH activity is the GH antagonist, pegvisomant. The discovery of pegvisomant by Kopchick and Chen provides for a safe and efficacious compound for treating acromegaly by means of daily injections (72–74). The potential of pegvisomant in human cancers was illustrated in studies on the growth and progression of xenografts of COLO-205 human colon cancer cells in athymic nude mice; pegvisomant treatment had a 39% decrease in tumor-volume and a 44% decrease in tumor weight compared to controls (75). Recently, a novel 15-aminoacid long peptide antagonist, S1H, of the GHR was developed that inhibits hGH-mediated STAT5 phosphorylation in cultured cells (76). However, both pegvisomant and S1H lack the autocrine functions that predict effective cancer treatments.

BM001 will also allow studies on the factors that can influence IGF1 levels. Mainly controlled by GH, also (upstream-acting) ghrelin, sex steroids, glucocorticoids, cytokines, and in particular the nutritional status can affect IGF1 levels (4, 46). As prolonged drug treatment will probably be needed to keep IGF1 levels sufficiently low to prevent tumor reappearance or to target longevity, BM001 will be a useful tool to investigate the ancillary factors that affect IGF1 levels. Based on studies on GH insensitive humans and mice, possible deleterious effects might be: a higher percentage body-fat, obesity, glucose intolerance, hepatic steatosis, and insulin resistance.

In conclusion, we have identified a set of small molecules that reduces both endo- and autocrine activities of the GH/IGF1 axis with the potential to inhibit GH-driven cancer growth and anti-cancer drug resistance. We envisage that BM001 may also be implemented in the control of imbalanced metabolic conditions as in diabetes, obesity, and impaired cognition in a variety of species.

## Data availability statement

The original contributions presented in the study are included in the article/**Supplementary Material**. Further inquiries can be directed to the corresponding authors.

## Ethics statement

All experiments were performed in accordance to international guidelinesand approved by Experimental Animal Committee Utrecht to Dr. Derksen (DEC-Utrecht, University Utrecht, Utrecht, The Netherlands).

## Author contributions

Conceptualization: GS, JM, LV. Funding acquisition: GS, JK. Investigation: LV, AM, FM, EV, PM, ET-S, MA, PD, GS. Data curation: PM. Data analysis: LV, PM, DE. Methodology: GS, LV, DE, PD, JM. Writing, original draft: GS. All authors have read and agreed to the final draft of the manuscript. All authors contributed to the article and approved the submitted version.

## Funding

This work was supported by the Dutch Technology Foundation Stichting Technische Wetenschappen (STW), which is the applied science division of the Nederlandse Organisatie voor Wetenschappelijk Onderzoek (NWO), and the Technology Program of the Ministry of Economic Affairs, Grant 11155: “Targeting the Jak2-GH receptor interaction for treatment of cancer”, by the European Network of Excellence, RUBICON “Role of ubiquitin and ubiquitin-like modifiers in cellular regulation” (Grant LSHG-CT-2005-018683); the Marie Curie Network, “UbiRegulators” (Grant MRTNCT-2006-034555), the Dutch Cancer Society (KWF-UU-2014-7201 and KWF-UU-2016-10456).

## Acknowledgements

The authors thank Daphne Lelieveld and Romina Pagliero for helping with the Jak2-inducible γ2A cell line, and Wienand Omta for help with the data analysis, Dennis Piet, Sirik Deerenberg and Tom Speksnijder for the syntheses of BM001, Nanda Sprenkels for QC, formulation and solubility studies on BM001.

## Conflict of interest

Authors GS, PM and HV serve as scientific advisors and have equity in Bimini Biotech BV, a biotechnology company that seeks to exploit regulators of GH/IGF1 activity but does not provide financial support for the technology described in this paper. Author LV is employed by College ter Beoordeling van Geneesmiddelen. Author DE is employed by Core Life Analytics B.V.

The remaining authors declare that the research was conducted in the absence of any commercial or financial relationships that could be construed as a potential conflict of interest.

## Publisher’s note

All claims expressed in this article are solely those of the authors and do not necessarily represent those of their affiliated organizations, or those of the publisher, the editors and the reviewers. Any product that may be evaluated in this article, or claim that may be made by its manufacturer, is not guaranteed or endorsed by the publisher.
