# Supplementary material for: Small molecules to regulate the GH/IGF1 axis by inhibiting the growth hormone receptor synthesis
Source: Front Endocrinol (Lausanne). 2022 Jul 28;13:926210. doi: 10.3389/fendo.2022.926210 (PMC9365994; doi:10.3389/fendo.2022.926210)
Supplement: Supplementary file 1 [file DataSheet_1.pdf]

Supplementary data to Van der Velden et al.

1. Cell based primary screen

The cells

The assay was based on Fos-zippered GHR cytosolic tails (fos-GHRct) subsequently acting as Jak2 substrates and signaling platforms (Nespital et al., 2016; Sedek et al., 2014). For this

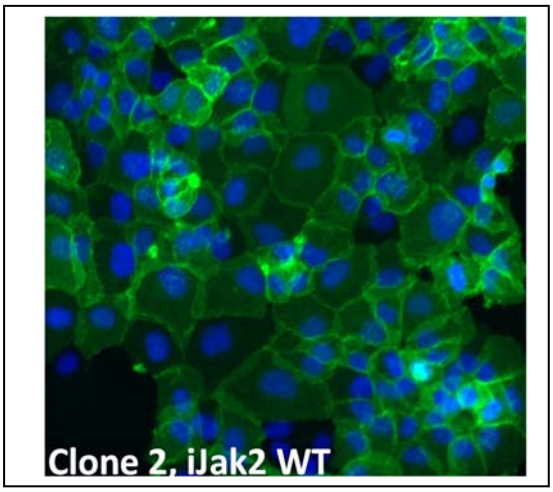

Fig S1A. Immune Fluorescence staining of γ2a monoclonal cell line stably transfected with an inducible Jak2 WT expression construct.

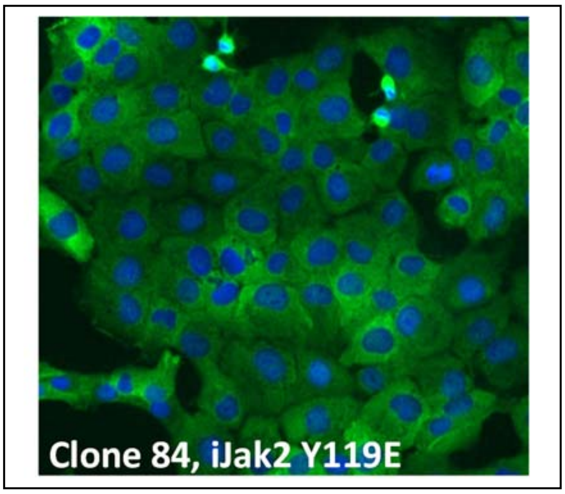

Fig S1B. Immune Fluorescence staining of γ2a monoclonal cell line stably transfected with an inducible Jak2 Y119E expression construct.

aim we used a human fibroblast cell line

γ2A, that does not express Jak2. From this line we generated doxycycline inducible Jak2 clones, expressing either wild type Jak2 or Y119E mutant Jak2 whose FERM domain does not interact with the box-1 sequence of the GHR (Putters et al., 2011). This difference is clearly visible in Fig. S1: While Jak2 (iJak2 WT) is mainly present at the cell surface, its binding-deficient mutant (iJak2 Y119E) distributes throughout the cytosol.

The principle

In Nespital et al., we showed that phosphorylated cytosolic tails are resistant to ubiquitination and proteasomal degradation (Nespital et al., 2016). To establish a sensitive test, sufficient Jak2 is needed. This was accomplished by using a doxycycline inducible promoter (Putters et al., 2011) and transient transfection of the fos-GHRct (Fig. S2). When fos-GHRct are co-expressed with Jak2, the levels of the phosphorylated fos-GHRct is increased (Fig. S3). To control the system, we used fos-GHRct mutated in box-1 or the cell line expressing mutant (Y119E) Jak2. The box-1 motif is the binding site of Jak2 in GHR; mutation of this motif (all four proline into alanine) is known to reduce the binding between GHR and Jak2. In the primary screen we measured 4 different signals: fos-GHRct (anti-GHR), tyrosine phosphorylated fos-GHRct (anti-pY), tyrosine phosphorylated Jak2 (anti-pY or anti-pY1007/1008) or pSTAT5 (anti-pSTAT5). Compounds that lower these signals, due to inhibition of either fos-GHRct stabilization or fos-GHRct synthesis, are considered positive.

Cell based GHR-Jak2 assay principle

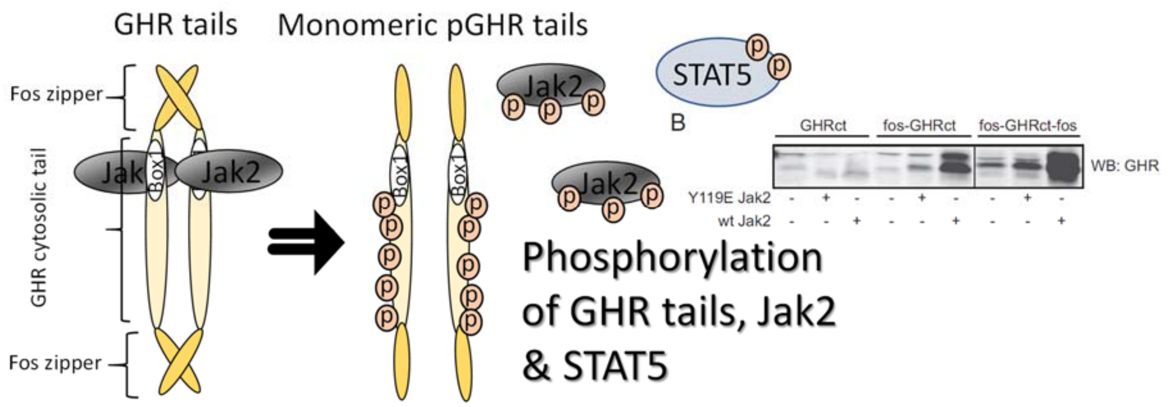

Fig S2. Co-expression of fos-GHRct (wild type) and Jak2 (wild type) in γ2A cells results in accumulation of phosphorylated fos-GHRct and Jak2. For experimental proof see Fig S3.
B. Western blotting of lysates of Hek293 cells transfected with the GHRct-zipper constructs alone, cotransfected with either wild-type Jak2 or Jak2 containing the Y119E-binding mutation (Nespital et al., 2016; Sedek

The specificity of the assay is best illustrated in the lower panels of Fig. S3 (pY staining). Only if fos-GHRct are expressed and at the same time wtJak2 is induced, both pY-Jak2 and pY-GHRtails (lanes 3 and 14) show a strong pY label. The other lanes show the specificity: no or little label if the FERM domain of Jak2 is disabled (lanes 7-10), or if box-1 in the fos-GHRct is absent (lanes 13-14) or if Jak2 synthesis is not induced (dox-minus). Also, if GHRct (without fos-zippers), lanes 4-5, 15-16, were transfected, no pY label could be detected, in agreement with Nespital et al., (Nespital et al., 2016)). Quantification of fos-GHRct with anti-GHR (Fig. S3, upper panel) gives clear results, compare lanes 2 and 3, 7 and 8, 13 and 14.
.

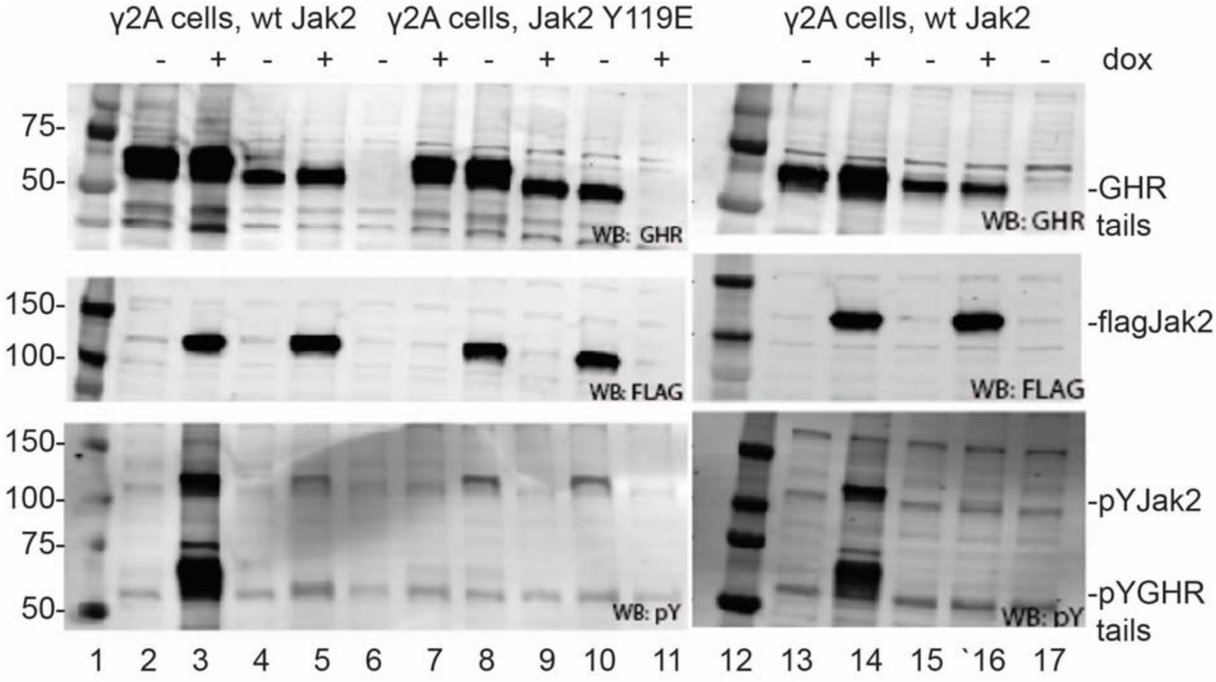

Fig S3. Immunoblots of total cell lysates from γ2A cells expressing inducible flag-tagged Jak2 constructs. Cells expressing either wild type Jak2 (lanes lanes 2-5) or Y119E-mutant-Jak2 (lanes 7-10) were transfected with fos-GHRct (lanes 2 and 3) or unzippered GHRct (lanes 4, 5, 15, 16). In the right panel fos-GHRct were expressed with an intact box-1 (lanes 13-14) or with box-1 deleted (lanes 15-16). Expression of Jak2 proteins (wild type or mutant) was induced by the addition of doxycycline (+ dox). Lanes 1 and 12, molecular weight standards; in lanes 6, 11 and 17 the cells were transfected with a GFP construct as controls.

Immunoblots are not appropriate for high throughput screening. Using fos-GHRct-transfected cells as in Fig. S3 and pY-specific primary antibodies, we developed a high throughput screening assay based on pY-fos-GHRct immune fluorescent detection in 384-well plates with automated microscopy. Together with Fig. S3, Fig. S4 illustrates the specificity of the immune label of fos-GHRct and pY-fos-GHRct. fos-GHRct. pY-positive cells are only visible if they are transfected with fos-GHRct. Untransfected cells show neither GHR nor pY signal.

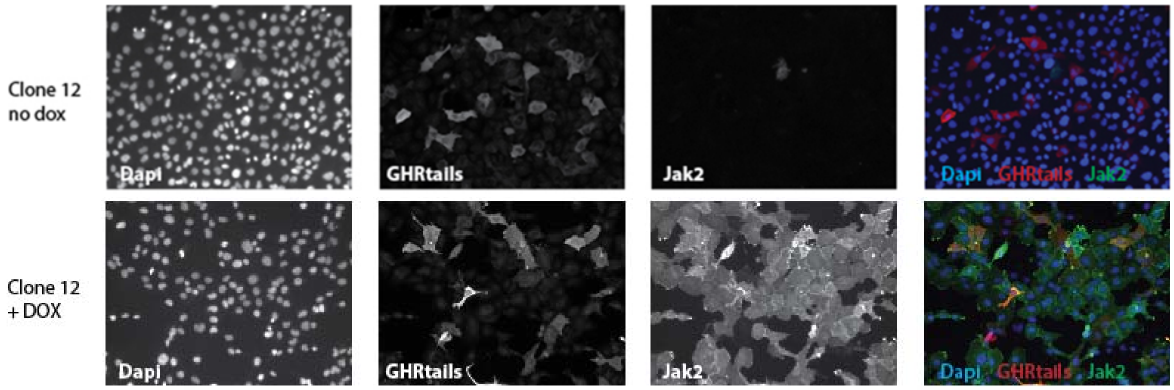

Fig S4A. Immune fluorescent staining of γ2A cells (clone 12) with no doxycycline added and no Jak2 expression (upper panel), or treated with doxycycline to induce the expression of Jak2 protein (lower panel). From left to right: Dapi staining to visualize nuclei, fos-GHRct staining: GHRtails are detected by rabbit polyclonal anti serum against GHR, Jak2 staining: Jak2 proteins are detected by an anti-Jak2 commercially available mouse monoclonal antibody. Overlay: Blue, Dapi; red, GHR; green, flagJak2.

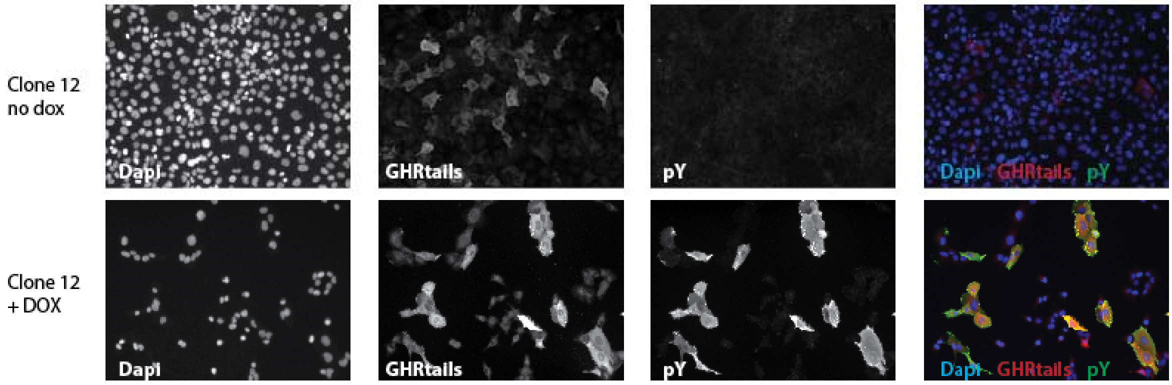

Fig S4B. Immune fluorescent staining of γ2A cells (clone 12) with no doxycycline added and no Jak2 expression (upper panel), or treated with doxycycline to induce the expression of Jak2 protein (lower panel). From left to right: Dapi staining to visualize nuclei, fos-GHRct staining: GHRtails are detected by a homemade rabbit polyclonal anti serum against GHR, phosphotyrosine (pY) staining: proteins with phosphotyrosine groups are detected by an anti-pY commercially available mouse monoclonal antibody. Overlay: Blue, Dapi; red, GHR, green, phosphotyrosine (pY).

Using the HCS explorer software with the Cell Insight automated microscope (Thermo Fisher) (Omta et al., 2016) we developed a protocol that automatically identifies GHR positive cells in a microscopic picture. This enables automatic analysis of the percentage of cells that are positive for the phosphotyrosine (pY) signal.

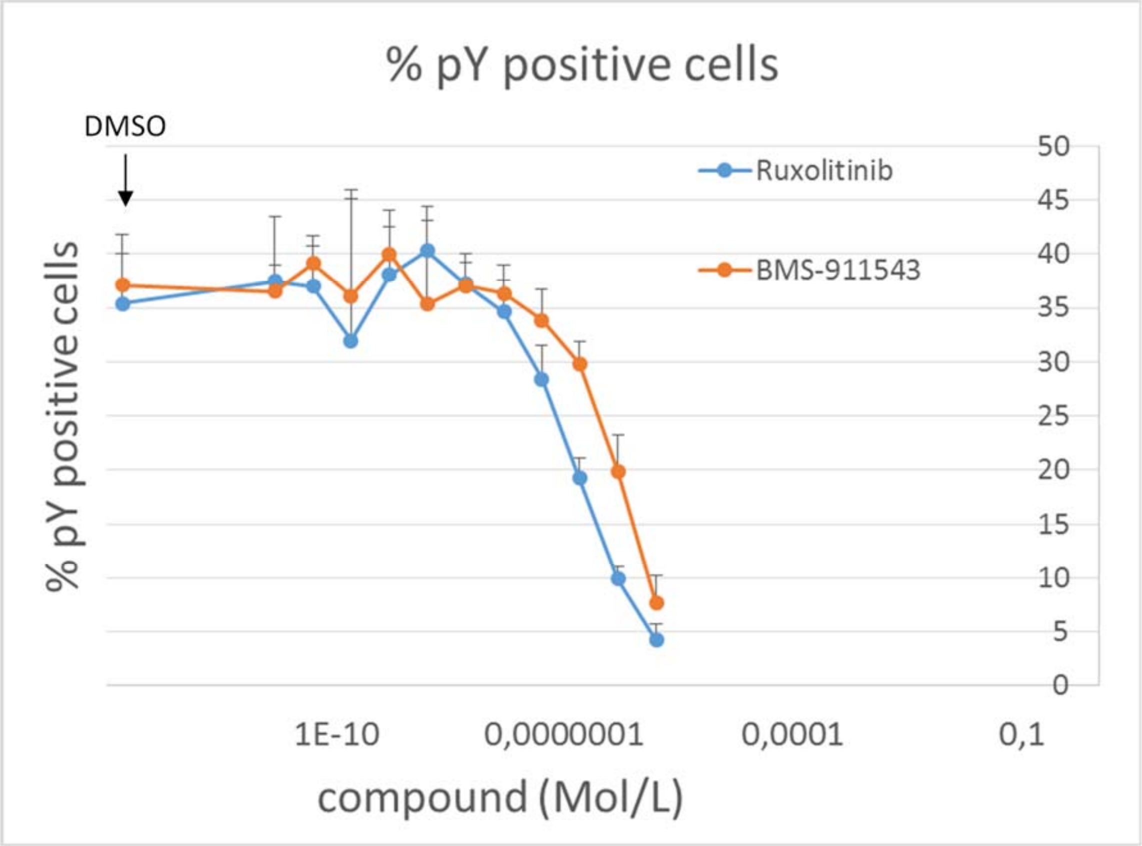

Fig S5. Percentage phosphotyrosine (pY) positive cells. fos-GHRct-transfected γ2A iJak2 cells were seeded in a 384-well plate. After 20 h the inhibitors were added and after 4 h Jak2 expression was induced by adding doxycycline. The cells were incubated for an additional 24 h, fixed, and the next day, stained with anti-pY antibodies. To cover one whole well in a 384 well plate, 4 microscopic fields were taken per well and analyzed. The number of pY-positive cells were identified using the HCS software and expressed as a percentage of total cells. The IC50 was in the nano-molar range for both Jak inhibitors.

Using this setup we first tested if we could see a reduction in phosphotyrosine signal using two known Jak2 inhibitors; Ruxolitinib, a FDA approved drug for the treatment of myelo-fibrosis, which targets both Jak2 and Jak1 and BMS-911543, which targets Jak2 and is in clinical trial for the treatment of myelofibrosis. Fig S5 shows co-expression of fos-GHRct with Jak2 induces pY-label in 37% of the cells (indicating the transfection efficiency). Both inhibitors decrease the pY signal in these γ2A cells treated for 24 h with Ruxolitinib and BMS-911543 with IC50 values in the nanomolar range. We decided to use Ruxolitinib as a positive control in our screening assay.

Based on these data, we fully automated the in-cell detection protocol (compound addition and fos-GHRtail+pY staining procedures) on our Sciclone robot to a 5-day protocol (primary screen):

- Day 1: Seed γ2A iJak2 cells in a 10 cm dish
- Day 2: Transfect cells with fos-GHRct construct using Lipofectamine2000. After at least 4 h, trypsinize the cells, count and reseed in blackwell clear bottom 384 well plates
- Day 3: Add the compounds of the SPECS library and the controls (negative control = only DMSO, positive control = 10μM Ruxolitinib) to the cells with the robot. After 2-3 h, add doxycycline to induce the expression of Jak2 protein
- Day 4: 20 h after doxycycline addition, fix cells with paraformaldehyde, store in PBS at 4°C.

- Day 5: Stain fos-GHRct and pY signal using specific primary and secondary antibodies. Nuclei were stained with DAPI. The full staining protocol is automated with the robot. After staining, 4 microscopic images of individual wells of the whole 384 well plate were immediately taken using the automated microscope (Cell Insight, Thermo Fisher). All information regarding the microscopic read out of the 384 well plates was stored on our servers for data analysis.

In total we screened 38,480 SPECS compounds (121 plates, reformatted to 384 well) in duplicate. Each plate contained 320 wells with compound, 64 wells on each plate were used for positive and negative controls.

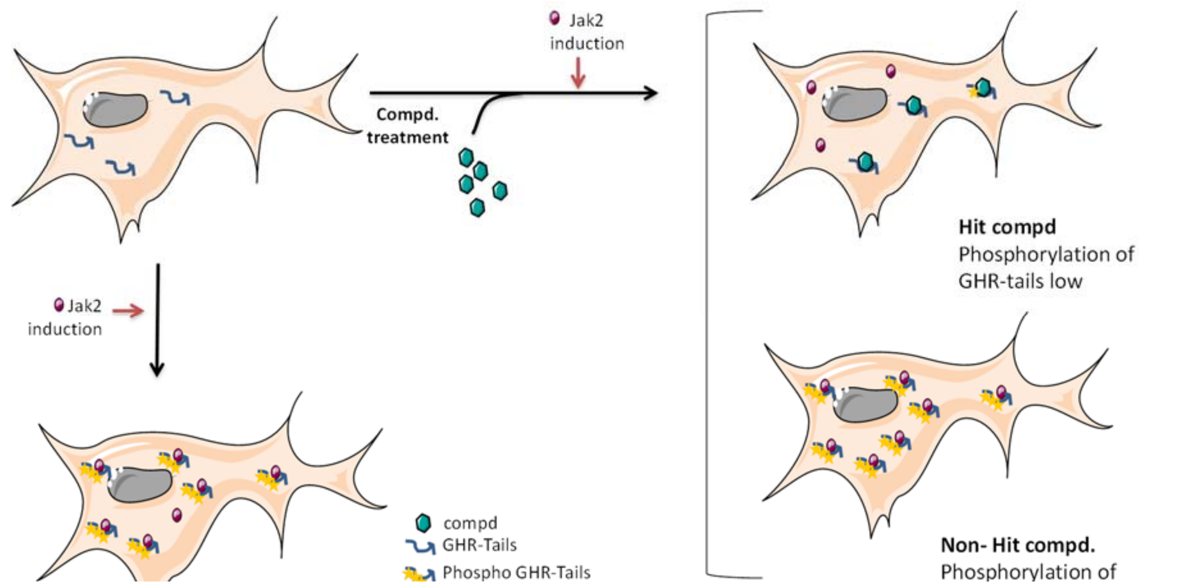

Fig S6. Cartoon of primary screen read-out to identify compounds that lower the levels of fos-GHRct and pY-labeled species (fos-GHRct and Jak2). When the γ2A iJak2 cells express fos-GHRct in the absence of Jak2 there is a relatively low concentration of tails in the cell and these tails are not phosphorylated. Upon induction of Jak2 in these cells, fos-GHRct and Jak2 form a protein complex and Jak2 phosphorylates fos-GHRct on tyrosine residues. A “hit-compound” is identified when Jak2 is no longer able to bind to and phosphorylate the fos-GHRct or either of the two interactors is decreased/absent. Also (general) kinase inhibitors will inhibit the phosphorylation of tyrosine residues in the fos-GHRct. Similarly, activation of tyrosine phosphatases will reduce the pY signals.

2. SPECs library quality control and re-formatting

To ascertain the quality of the SPECS library that was stored and previously used for other applications in the lab, we performed a quality control check.
The plates were first inspected visually and separated in three classes:

1. Good plates, which seem proper sealed with no visible leakage
2. Suspicious plates, somewhat leaky, restricted to liquids at the sealing caps
3. Bad plates with a lot of leakage and with badly attached or completely loose caps

The plates classified as “Bad” were discarded (16% of the total library). The rest of the plates were sampled for chemical integrity analysis. 50 samples of classes 1 and 2 were analyzed by SPECS on LC-MS. The results indicated that at least 75% of the samples showed a purity of more than 90%. We decided these plates, (84% of the library) of sufficient quality to use for the primary screen. The plates were re-formatted into 384-well plates and stored in containers with 100% nitrogen (N_2) at room temperature.

3. Screening results

In total we screened 121 384-well plates. On each plate contained 320 wells with compound, and 64 wells with positive and negative controls. In total we screened 320x121 plates =

38480 SPECS compounds that could lower the levels of phosphorylated fos-GHRct and pJak2. The whole screen was performed in duplicate.
For data analysis we used two software packages. Four microscopic pictures of each individual well in all 384 well cell assay plates were taken with a “Cell Insight” (Thermofisher) automated microscope. Using the accompanying software of the Cell insight, HCS explorer, we identified all the cells in a well using the DAPI stain (valid object count). Cells that were on the edge of the picture, dividing cells or with aberrant large or small nuclei were excluded from further analysis. Thereafter, cells which stain positively for GHR and pY were selected. The software calculates “cell level” data and “well level” data.
Next, we imported the “well level” data from HCS explorer into the StratomineR Software package. Stratominer is an in-house developed software program that is suited for high content data analysis (Omta et al., 2016).
Process of data analysis in Stratominer:

1. All the well parameters values were extracted from HCS Explorer
2. All the MEAN parameters + validobjectcount were selected for further analysis
3. A normalization using the median of the negative controls was applied on the parameters (except validobjectcount)
4. The normalized parameter plus the raw validobjectcount were included for factor analysis
   - Six factors were retained where validobjectcount was not really contributing to the determination coefficient (explained variance of the dataset)
   - Factor 2 (fos-GHRct) and Factor 6 (pY) were selected for generating a hitlist
5. The hitlist was created by calculating the Manhattan distance of factor 2 + factor 6 against the median of the negative controls of each plate
   - The p-value in the list is the average of the two p-values on both replicates for each compound/control
   - Next to that the normalized raw parameter avgintch2 (pY signal) + avgintch3 (fos-GHRct signal) and the raw unnormalized parameter validobjectcount is added (all averages of both replicates for each compound/control)

The number of hits with a combined p-value of smaller than 0.1 was 4402, including the controls. The combined p-value is the average of the two p-values in each duplicate. Of those 4402, 3224 hits belong to the category “control”, and 1178 hits belong to the category “sample”.

Values smaller than 1 in the parameter “combined_WELL_CellHealthProfilingV4WellMEAN_-TargetAvgIntenCh2” were considered hits with down regulated pY signal. In total there were 376 hits that fall in the category down regulated based on pY signal. The category upregulated pY signal amounted 802 hits This included for example compounds that are autofluorescent in the green (pY) channel (λ 488 nm).
Of the down regulated hits, some well numbers (positions) seem systematically to be overrepresented. Probably these are false positive “hits” due to a robotics (e.g. washing with aquamax) during the actual screening. Well numbers that turn up 5-times or more as frequent ‘wells’ were discarded, mostly at the outer edges of the plates: A17, A22, B22, O06, O20, O21, P03, P04, P05*, P06, P12, P20, P21 and P22. After correction for this the primary screen resulted in 376-219 = 157 hits with downregulated pY signal and p-val < 0.1 The 157 hits with downregulated pY signal were given “red flags” if the same well number turns up in consecutive plate numbers (the first plate doesn’t get a flag, the next one after that does), or if the valid object count is relatively low in relation to the actual physical position in the 384 well plate. (wells on the edges of the plate have lower cell numbers and lower valid object count numbers than wells in the middle of the plate). In total 42 red flags were given based on these criteria. Corrected for this, the screen resulted in 115 hits (157-42) with down-regulated pY signal and p<0.1 (wells on the edges of the plate have lower cell numbers and lower valid object count numbers than wells in the middle of the plate). A last correction is needed for autofluorescence in the red channel (fos-GHRct signal, λ > 568 nm), and the presence of compounds registered in the literature as “frequent hitter(s)”.

4. Identification of lead compounds.

Of the 115 hits we selected 109 compounds for hit confirmation experiments. One set of compounds was re-tested in the primary screen. This resulted in the confirmation of 10 hits, (Fig. S7 and Table S1). They were shipped to Dr. Fabrice Malergue, and Andreas van Agthoven at Beckman coulter Immunotech in Marseille to be tested in a FACS assay for the GHR-dependent activation of STAT5 (Malergue et al., 2015). In short: A mixture of blood and IM9 human lymphoblast was incubated with the drugs (50 µM) for 15 MIN; GH and GM-CSF were added 7 min before the cells were fixed with formaldehyde. Blood cells are sensitive to GM-CSF, and not to GH; IM9 cells are sensitive to GH. A mixture of three antibodies in three colors; CD14-FITC, CD34-PE and p-Stat5-Alexa 647 was used to analyze the p-Stat5 levels of monocytes and IM9 cells. Cross referencing the hits in these two assays (>30% inhibition in the FACS assay and the re-testing in the primary screen) yielded a list of 4 hits (BM004, BM012, AQ-390/41647621, and AE-413/30061025 in Table S1). SPECS delivered a fresh set of compounds of the 17 best hits and the FACS assay was repeated in Marseille (Fig. S8). Only drugs BM004 and BM012 decreased the GH-induced Stat5 signaling (Fig. S8A); none of the drugs inhibited GM-CSF-induced Stat5 signaling (Fig. S8B).

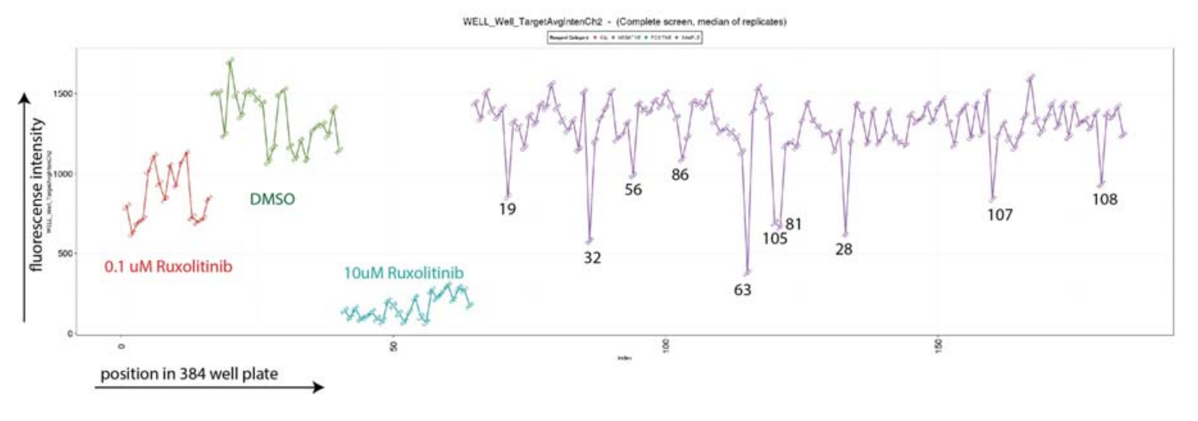

Fig. S7. Confirmation run of primary screen where we read out phospho tyrosine (pY) status of fos-GHRct in γ2A cells with a specific pY-antibody which is detected by a fluorescent tagged secondary antibody (y-axis). Negative control in green, cells treated with DMSO. Positive controls in red and blue, cells treated with 0.1 and 10 µM respectively. Hit conformation samples in purple. The mean of 6 replicate runs is shown.

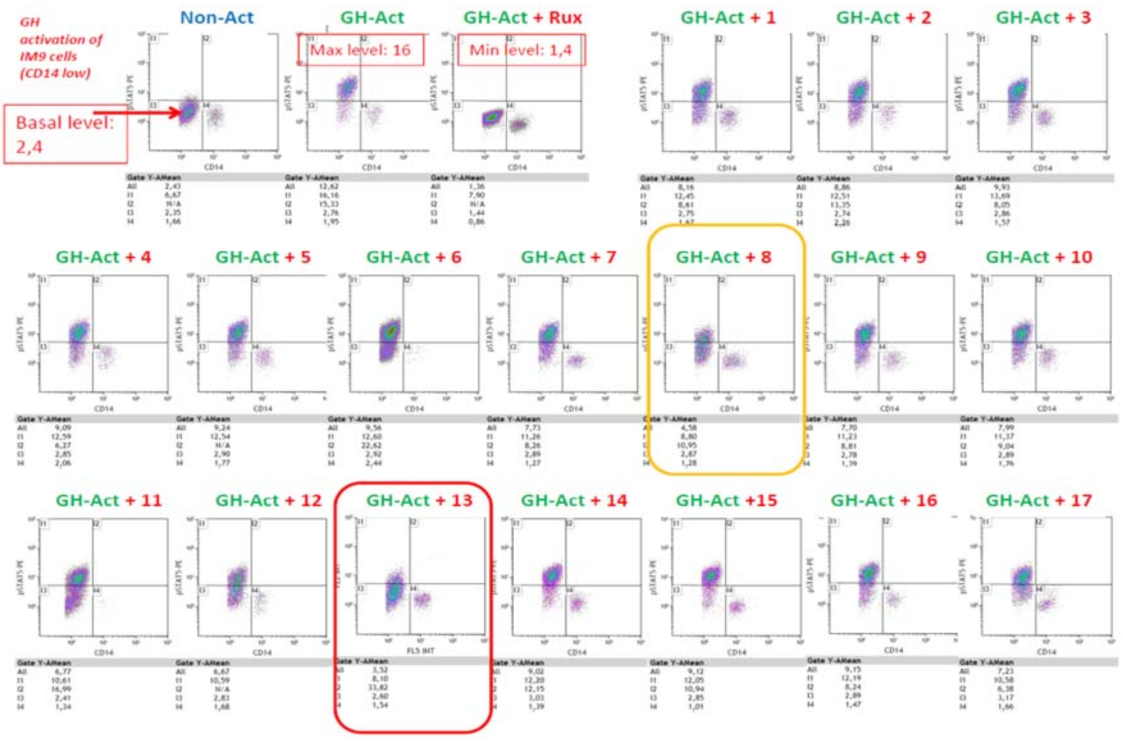

Fig. S8A. FACS plots of compounds that inhibit GH-induced GHR-Jak2-Stat5 signaling in IM9 lymphoblasts. The end point of the signaling cascade is measured by assessing the phosphorylation status of STAT5. Compounds BM012 (yellow box number 8) and BM004 (red box number 13) inhibit the phosphorylation of Stat5. Conc of drugs: 1 mM; incubation time 30 min

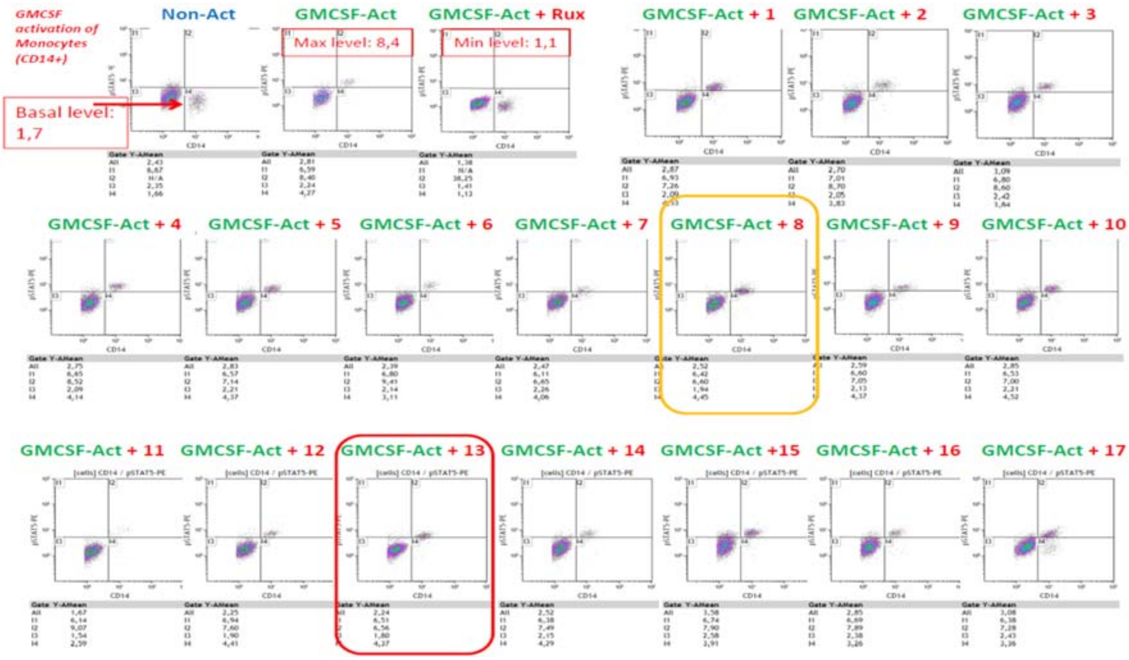

Fig. S8B. FACS plots of compounds that inhibit GM-CSF-Jak2-Stat5 signaling in blood monocytes. The end point of the signaling cascade is measured by assessing the phosphorylation status of STAT5. Compounds BM012 (yellow box number 8) and BM004 (red box number 13) have no effect on the phosphorylation of Stat5. Conc of drugs: 1 mM; incubation time 30 min

hitnumber	well # hit confirmation	wellSPECSplate	SPECS runnumber	SPECS plate barcode	SPECS compound
19	C17	C08	RW032412	93000300615010	AK-968/40318665
32	E06	D11	RW032629	93000300618820	BM004
56	E22	B10	RW034950	9300030063311	AG-690/11156170
86	F21	H04	RW031479	93000300603291	AG-690/11821553
63	H05	C11	WD200090	93000300458501	AF-399/13278014
105	H14	D09	WD200001	93000300451380	AE-413/30061025
81	H17	F05	RW032561	93000300616789	AQ-714/41674990
28	I21	G05	RW032403	93000300614747	AQ-390/41647621
107	L14	B10	WD200002	93000300451465	BM012
108	N14	G07	WD200075	93000300457306	AN-329/14225006

Table S1. The 10 best compounds after retest in primary screen

Taken together, we identified two lead compounds, BM004 and BM012 that decrease the levels of tyrosine phosphorylation of fos-GHRct and that of STAT5. They act very rapidly (within 30 min) and are specific for the Jak2/STAT5 pathway via GH which suggest that they act upstream in the GHR pathway. **The compounds did not affect the GM-CSF pathway** (see Fig. S8B). They share a common chemical structure. Compound BM012 belongs to the class of quinazolines for which many patents are registered as anti-cancer compounds which inhibit tyrosine kinases

5. Characterization of the 2 lead compounds

Through the primary assay we identified two lead compounds that inhibit Jak2-mediated phosphorylation of fos-GHRct. Next, we assessed whether they also act on the GH-Jak2-STAT5 signaling pathway.

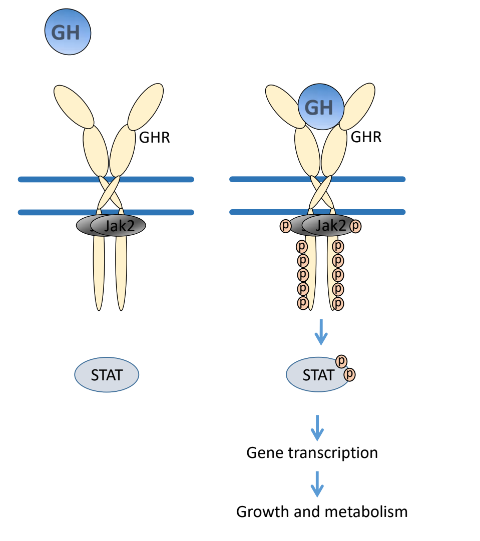

Fig. S9. Growth Hormone signaling pathway. Growth hormone binds to the GHR and induces a conformational change in the cytosolic tails of the receptor. This conformational change leads to the activation of the two Jak2 molecules, which phosphorylate each other. The activated Jak2 also phosphorylates tyrosine residues in the cytosolic tails of the GHR and phosphorylates the transcriptional activator STAT5.

To address this question, we transfected the fos-GHRct construct in γ2A cells, described in Fig. S1, and according to the protocol of the primary screen. For analysis we used antibodies against activated Jak2 (pY at position Tyr-1007/1008) and activated phosphorylated STAT5 (pSTAT5). The concentration courses are depicted in Fig. S10. Both lead compounds decreased the phosphorylation signals of fos-GHRct (pY), of the active site of Jak2 (p1007/1008) and of STAT5. The IC50 of BM004 was comparable to that of Ruxolitinib (IC50 ~ 0.3 μM, in agreement with Furqan et al. (Furqan et al., 2013).

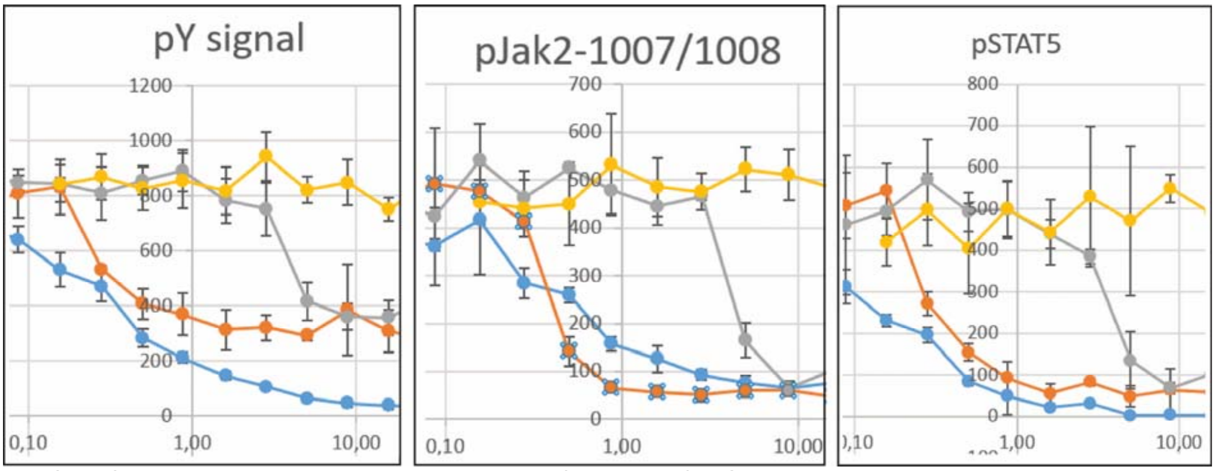

Fig. S10. Concentration curves: γ2A cells were transfected with fos-GHRct and Jak2 expression was induced with doxycycline. Cells were treated with the compounds for 20 h. The concentrations (x-axis) are indicated in µM. pY levels were assessed by quantifying the phosphorylation status of fos-GHRct (left panel)), of pJak2-1007/1008 (middle panel) and of pSTAT5 (right panel). Ruxolitinib, blue; BM004, orange; BM012, grey; DMSO, yellow.

Next, we assessed if the compounds also inhibit GHR signaling of full length GHR. Hek293 cells were co-transfected with a rabbit pcDNA-GHR and with Jak2, and incubated with increasing concentrations of the 2 compounds or with Ruxolitinib (10 µM). Cells were then stimulated with GH for 15 min and the GHR signaling activation was assessed by immunoblotting for pY of the GHR (Fig. S11). Both lead compounds reduced the levels of tyrosine phosphorylated full length GHR, stimulated with GH, concentration-dependent.

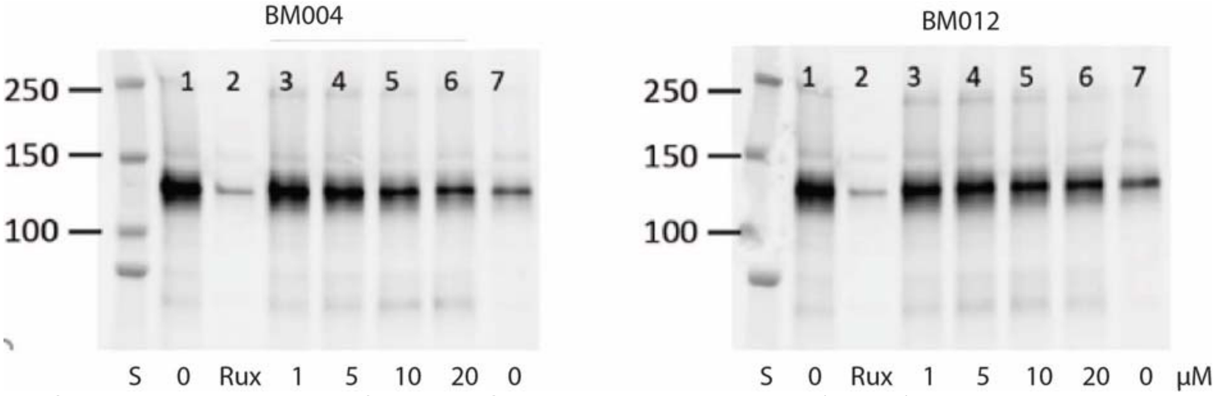

Fig. S11. Hek293 cells were transfected with GHR and Jak2 or JAK2 Y119E (lanes 7), and treated with the compounds for 20 hours. The amounts of labeled GHR were assessed by quantifying the phosphorylation status of GHR.

6. The second round of analogs

SPECS Compound Handling B.V. provided 109 new analogs of BM004 and BM012. They were tested in the primary assay, and we identified **21 new compounds** that lowered the

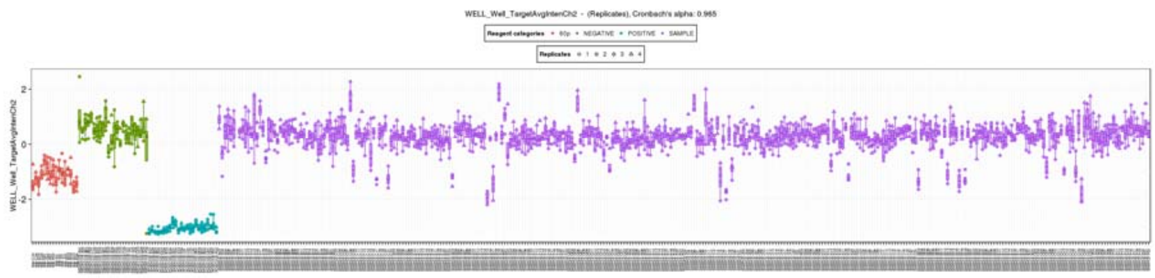

Fig. S12. X-axis: position in 384 well plate, Y-axis: pY fluorescence intensity. Purple data points, analogs; blue data points, 10 µM Ruxolitinib; pink data points = 1 µM Ruxolitinib; green data point, DMSO. Data points are represented as box plots of 4 replicates.

fos-GHRct phosphorylation (Fig. S12), a remarkable hit rate of 17%. Next, we determined the dose-response effect (IC50) of these 21 new compounds on pY-levels of fos-GHRct. The compounds were divided in four categories: no dose-response effect (6), effect less than Ruxolitinib (9), similar to Ruxolitinib (3) and better than Ruxolitinib (3), (Fig. S13). None of the compounds reduced the pY signal as much as Ruxolitinib (close to zero). The compounds all plateaued at 200 units of relative fluorescence intensity.

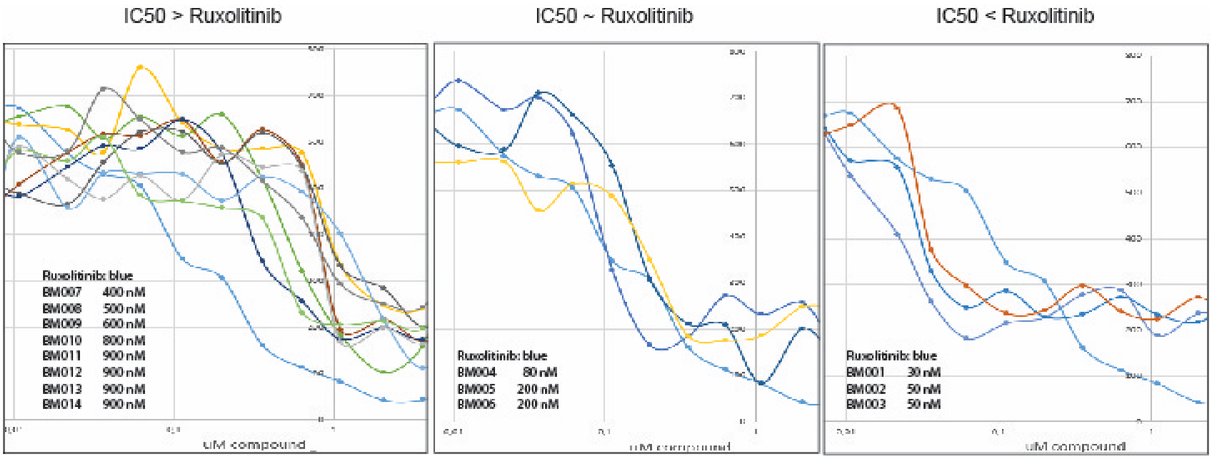

Fig. S13. Dose-response curves of the 14 compounds with IC50 values below 1 μM, according to primary screen protocol. They are depicted in 3 panels as shown at the top. Ruxolitinib served as markers. Ruxolitinib inhibits Jaks, has no effect on cell proliferation.

7. Cell proliferation assay

Next, we assessed the effect of the best 5 lead compounds (BM001-BM005) for the inhibition of cell proliferation/induction or cell death. We used the CyQuant assay of Molecular Probes, which stains only the nuclei of living viable cells. With a standard green filter set the fluorescence from the nuclei is quantified in a multi-well plate spectrophotometer. All 5 compounds reduced the amount of cell numbers when incubated for 24,48 or 72 h. Fig. S14 shows the data points of the a 48-h incubation of the MDA-MM-231 and Colo-205 cell line, respectively. Data points for 24h and 72 h show a reduction of cell numbers to respectively 80% and 20-40%, respectively (not shown), but at the higher compound concentrations. As for the dose-response curves (Fig. S13), the compounds show an equal off-set effect of ~50% for the MDA-MM-231 cells, which is absent in the Colo205 cells. Apparently, the cell line is not homogeneous and may contain insensitive cells. The observation indicates that the compounds affect cell proliferation.

We chose the breast cancer cell line MDA-MM-231, as an appropriate model for Triple Negative Breast Cancer (TNBC) studies to be used in mice in the future. We also included the Colo-205 cells, because in literature this cell line shows a GH-dependent growth effect upon tumor inoculation in mice. In a cell counting assay with addition of GH and compounds we did not see an additional effect of GH (data not shown). This is probably due to autocrine GH effects in cancer cells.

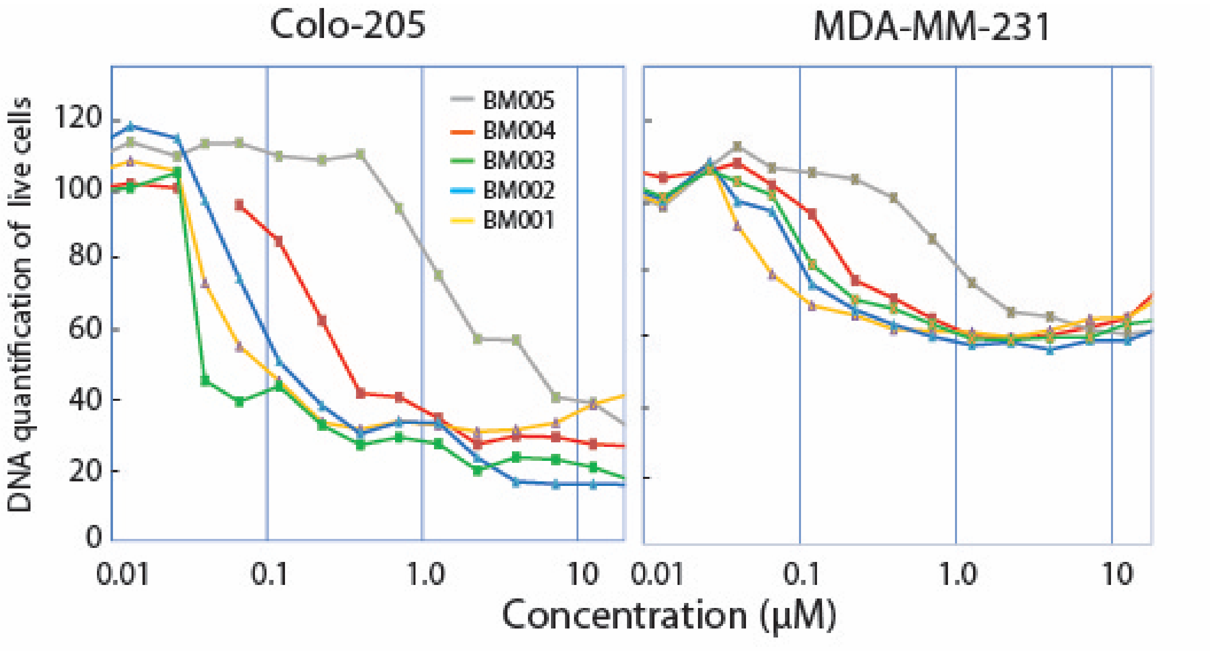

Figure S14. The best 5 compounds from the dose-response curves were used to test their effect on cell proliferation. They inhibit proliferation of triple negative human breast cancer (MDA-MB-231) cells and colon cancer cell (IC50 20-50 nM), with IC50 values in the same range as in the dose-response curves that measured pY values on fos-GHRct.

8. Effect of selected drugs on GH synthesis

To show that the compounds do not affect the synthesis of other proteins, we used the intracellular steady state concentration of a secretory glycoprotein (human growth hormone, hGH): its steady state is the result of synthesis, glycosylation, intracellular transport and secretion. Fig. S15 shows that only cycloheximide (a protein synthesis inhibitor) affected the amount of hGH, while none of the compounds changed the steady state concentration in the cells. This is an indication that the drugs do not affect the general protein synthesis

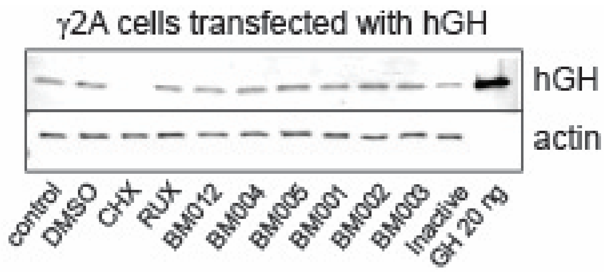

Figure S15. Y2A cells were transfected with 5 µg of a hGH-pcDNA construct in a 24-well plate; drug treatment overnight (10 µM) as indicated (CHX, cycloheximide 40 µg/ml). AG-690/11155024 (Specs) was used as inactive control drug, 10 µM. Right lane: human GH 20 ng (control). Only cycloheximide decreased the steady state concentration of hGH in the cells. hGH in the lysates was detected with a rabbit anti-GH antibody.

Furqan, M., N. Mukhi, B. Lee, and D. Liu. 2013. Dysregulation of JAK-STAT pathway in hematological malignancies and JAK inhibitors for clinical application. *Biomarker research*. 1:5.
Malergue, F., A. van Agthoven, C. Scifo, D. Egan, and G.J. Strous. 2015. Automation of a phospho-STAT5 staining procedure for flow cytometry for application in drug discovery. *J Biomol Screen*. 20:416-421.
Nespital, T., L.M. van der Velden, A. Mensinga, E.D. van der Vaart, and G.J. Strous. 2016. Fos-Zippered GH Receptor Cytosolic Tails Act as Jak2 Substrates and Signal Transducers. *Mol Endocrinol*. 30:290-301.
Omta, W.A., R.G. van Heesbeen, R.J. Pagliero, L.M. van der Velden, D. Lelieveld, M. Nellen, M. Kramer, M. Yeong, A.M. Saeidi, R.H. Medema, M. Spruit, S. Brinkkemper, J. Klumperman, and D.A. Egan. 2016. HC

StratoMineR: A Web-Based Tool for the Rapid Analysis of High-Content Datasets. *Assay Drug Dev Technol*. 14:439-452.

Putters, J., A.C. da Silva Almeida, P. van Kerkhof, A.G. van Rossum, A. Gracanin, and G.J. Strous. 2011. Jak2 is a negative regulator of ubiquitin-dependent endocytosis of the growth hormone receptor. *PLoS ONE*. 6:e14676.

Sedek, M., L.M. van der Velden, and G.J. Strous. 2014. Multimeric growth hormone receptor complexes serve as signaling platforms. *J. Biol. Chem.* 289:65-73.
